# GLP-1 Receptor Agonists in Type 2 Diabetes and Beyond – New Insights 2015

**DOI:** 10.17925/EE.2015.11.01.21

**Published:** 2015-04-11

**Authors:** Baptist Gallwitz

**Affiliations:** Deputy Chief, Department of Medicine IV; Head of the Outpatient Clinic for Diabetes and Endocrinology, Eberhard Karls University, Germany

**Keywords:** Type 2 diabetes, obesity, GLP-1 receptor agonists, albiglutide, dulaglutide, liraglutide

## Abstract

Glucagon-like peptide-1 receptor agonists (GLP-1 RAs) were introduced for type 2 diabetes therapy nearly 10 years ago, among them short-acting compounds on the basis of the GLP-1-like peptide exendin-4 (exenatide and lixisenatide) and a long-acting GLP-1 RA based on the human GLP-1 sequence (liraglutide). Recently, two novel long-acting GLP-1 RAs on the basis of human GLP-1 sequence, for once-weekly application, have been approved for therapy of type 2 diabetes. Additionally, liraglutide has been approved for treatment of obesity at a higher dose than that used for diabetes therapy. This mini-review gives a short overview of the novel long-acting GLP-1 RAs albiglutide and dulaglutide and also reviews the studies of liraglutide in treatment of obesity leading to its approval for this use. These studies were largely presented at the annual meeting of the European Association for the Study of Diabetes (EASD) in fall 2014.

Glucagon-like peptide-1 receptor agonists (GLP-1 RAs) were introduced for the therapy of type 2 diabetes about 10 years ago, with exenatide the first drug in the class. Natural GLP-1 is secreted post-prandially by the L-cells of the intestine. It stimulates insulin secretion and inhibits glucagon secretion in a glucose-dependent manner. In addition to having direct effects on glucose metabolism, GLP-1 also slows gastric emptying, acts as a neurotransmitter in hypothalamic nuclei stimulating satiety, lowers blood pressure and has a positive influence on beta cell mass in animal models with high beta cell turn-over. Because GLP-1 itself has a very short half-life, long-acting GLP-1 RAs have been designed to use GLP-1 action for type 2 diabetes therapy.^1,2^

Recently, two GLP-1 RAs for once-weekly injection were approved for the treatment of type 2 diabetes. Albiglutide (Eperzan^®^, GlaxoSmithKline Pharmaceuticals) and dulaglutide (Trulicity^®^, Lilly Pharmaceuticals) are based on the human GLP-1 peptide sequence, whereas exenatide for once-weekly use (Bydureon^®^, AstraZeneca Pharmaceuticals), introduced in 2012, is based on the sequence of exendin-4, a peptide with >50 % sequence homology to human GLP-1.^3–5^ Apart from this difference, exenatide for once-weekly use has a long pharmacological action thanks to galenic preparation, with a retarded and slow continuous release of exenatide from a suspension of soluble microspheres loaded with the compound.^6^

This review aims to give an overview of the recently published clinical study data on the novel long-acting GLP-1 RAs albiglutide and dulaglutide with a human GLP-1 ligand. In addition, recent data on the long-acting GLP-1 receptor agonist liraglutide, which also has a human GLP-1 sequence and is for treating obesity, are presented. Most of the clinical study data dealt with in this article were presented at the recent annual meeting of the European Association for the Study of Diabetes (EASD) 2014. These data were also prerequisites for the approval of albiglutide and dulaglutide for the treatment of type 2 diabetes with once-weekly injections. The indication for liraglutide was widened to additionally include the treatment of morbid obesity without type 2 diabetes.

## Albiglutide

Albiglutide is a molecule consisting of two copies of a 30-amino acid sequence of a human GLP-1 dimer genetically fused to human albumin. Degradation is inhibited by a single amino acid substitution within the GLP-1 fragment. Additionally, the fusion to albumin results in a longer halflife. Maximum concentrations of albiglutide are reached 3 to 5 days after a subcutaneous dose, and steady-state concentrations are achieved after 4 to 5 weeks of once-weekly administration.^7^

Albiglutide has been studied in a set of prospective randomized clinical studies at various stages of type 2 diabetes and in different combination therapies. An overview of the comparative clinical study programme with albiglutide is given in *Table 1*. In summary, the programme consisted of eight independent studies (HARMONY 1 to HARMONY 8) with the usual primary endpoint of glycated haemoglobin (HbA_1c_) reduction at 26, 32, 52 or 104 weeks. Overall, the trials involved more than 5,000 patients, including more than 2,000 patients on albiglutide, representing more than 7,500 patient-years of treatment. The mean baseline HbA_1c_ for the study population ranged from 8.0 % to 8.5 %, and the mean baseline body weight was 83–95 kg.^8–16^

**Table 1:** Glycaemic Efficacy and Body Weight Changes in the Phase III Clinical Studies with Albiglutide (HARMONY Studies; summarised from references 8–16)

Study (Reference)	Comparator and Treatment Arms (Number of Patients)	Duration (Weeks)	Baseline HbA_1c_ (%)	Change in HbA_1c_ (%)	Baseline Body Weight (kg)	Change in Body Weight (kg)	Patients Needing Rescue Therapy (%)
HARMONY 1 Reusch et al. 2014^9^	ALB+PIO±MET (149)	52	8.1	−0.81*	98	0.3	24.4*
	PBO+PIO±MET (150)		8.1	−0.05	100	0.5	47.7
	ALB dose 30 mg						
	MET dose ≥1,500 mg						
	PIO dose ≥30 mg						
HARMONY 2 Nauck et al. 2013^10^	ALB 30 mg (100)	52	8.1	−0.7*	–	−0.7	20
	ALB 50 mg (97)		8.2	−0.89*	–	−0.9	16
	PBO (99)		8.0	0.15	–	−0.7	50
HARMONY 3 Ahren et al. 2014^11^	ALB+MET (297)	104	8.1	−0.63*^	90	−1.2**	25.8*^‖^
	SIT+MET (300)		8.1	−0.28	90	−0.9	36.4
	GLM+MET (302)		8.1	−0.36	92	1.2	32.7
	PBO+MET (100)		8.1	0.27	92	−1.0	59.2
	MET dose ≥1,500 mg						
	SIT dose 100 mg						
	GLM dose 2–4 mg (mean 3.1 mg)						
	ALB dose 30–50 mg (mean 40.5 mg)						
HARMONY 4 Weissmann et al. 2014^12^	ALB+MET±SU (496)	52	8.3	−0.67^§^	95	−1.1^	25.6
	GLA+MET±SU (239)		8.4	−0.79	95	1.6	23.8
	MET dose ≥1,500 mg (82 % on MET+SU)						
	ALB dose 30–50 mg (67 % on 50 mg)						
	GLA dose titrated per protocol (mean 35 U)						
HARMONY 5 Home et al. 2014^13^	ALB+MET+GLM (269)	52	8.2	−0.55*^‖^	91	−0.4**	n/a
	PIO+MET+GLM (273)		8.3	−0.80	91	4.4	
	PBO+MET+GLM (115)		8.3	0.33	90	−0.4	
	MET dose ≥1,500 mg						
	GLM dose 4 mg						
	ALB dose 30–50 mg						
	(70.1 % on 50 mg)						
	PIO dose 30–45 mg (54.9 % on 45 mg)						
HARMONY 6 Rosenstock et al. 2014^14^	ALB+GLA±OAD (282)	26	8.5	−0.82§	93	−0.7^	22.9
	Lispro+GLA±OAD (281)		8.4	−0.66	92	0.8	24.4
	Baseline OADs allowed: PIO, MET, AGI						
	68 % met only; 6 % MET+PIO; 23 % neither						
	GLA titrated per protocol (mean 53.2 cU)						
	ALB 30–50 mg (51 % on 50 mg)						
	Lispro adjusted per protocol (mean 50.6 cU)						
HARMONY 7 Pratley et al. 2014^15^	ALB+≥1 OAD (402)	32	8.2	−0.78^$^	92	−0.6	15
	Lira+≥1 OAD (403)		8.2	−0.99	93	−2.2^¶^	8
	Baseline OADs allowed: MET, TZD, SU						
	~40 % monotherapy, ~50 % two drugs						
	ALB titrated to 50 mg						
	Lira titrated to 1.8 mg						
HARMONY 8 Leiter et al. 2014^16^	ALB±OAD (246)	26	8.1	−0.83^	83.3	−0.79^	6.1^
	SIT±OAD (240)		8.2	-0.52	82.8	-0.19	12.1
	ALB 30–50 mg (mean 42.4 mg)						
	SIT dose based on renal function						

AGI = alpha-glucosidase inhibitors; ALB = aibigiutide; GLA = insulin giargine; GLM = glimepiride; Lira = liraglutide; MET = metformin; OAD = oral anti-diabetic agent; PBO = placebo; PIO = pioglitazone; SIT = sitagliptin; *ALB significant versus PBO; ^ALB significant versus active comparator; **ALB significant versus GLM; ^װ^ALB significant versus SIT; ^§^ALB noninferior to active comparator; ^$^Non-inferiority of ALB versus active comparator not met; ^¶^Comparator significant versus ALB.

An HbA_1c_ reduction of -0.55 % to -0.9 % was observed in these studies, and albiglutide was found superior to treatment using a sulphonylurea, pioglitazone or dipeptidyl peptidase-4 (DPP4) inhibitor. Non-inferiority was demonstrated versus insulin therapy.^3^ The mean body weight change was lower in comparison to other GLP-1 RAs, amounting to 0.3 to -1.2 kg, and was less when albiglutide was combined with oral anti-diabetics known to cause weight gain (pioglitazone, sulphonylurea).^8–16^

The overall hypoglycaemia rates were low, similar to those of other GLP-1 RAs, when albiglutide was used as monotherapy or in combination with metformin or pioglitazone. Higher rates were observed only in combination with a sulphonylurea or insulin. As expected when using a GLP-1 RA, the most commonly reported adverse events were gastrointestinal and occurred at a higher rate than with placebo, pioglitazone, sulphonylurea, a DPP-4 inhibitor or insulin, but less frequently than with liraglutide.^8–16^

**Table 2:** Glycaemic Efficacy and Body Weight Changes in the Phase III Clinical Studies with Dulaglutide (AWARD Studies; summarized from references 18–24)

Study (Reference)	Comparator and Treatment Arms (Number of Patients)	Duration (weeks)	Baseline HbA_1c_ (%)	Change in HbA_1c_ (%)	Baseline Body Weight (kg)	Change in Body Weight (kg)
AWARD 1 Wysham et al. 2014^18^	DU 0.75+PIO+MET (263)	26	8.1	−1.30^*	96	0.2
	DU 1.5+PIO+MET (260)		8.1	−1.51^*	96	-1.3
	EXE 10+PIO+MET (252)		8.1	−0.99*	97	−1.07
	PBO+PIO+MET (124)		8.1	−0.46	94	1.24
	MET dose ≥1,500 mg					
	PIO dose ≥30 mg					
AWARD 2 Giorgino et al. 2014^19^	DU 0.75 mg (272)	52	8.1	−0.76^§^	86.3	−1.33^§^
	DU 1.50 mg (273)		(average for all groups)	−1.08^	(average for all groups)	−1.87^§^
	GLA (262)			−0.63		1.44
	MET dose ≥1,500 mg					
	Average GLA dose 29.4 U					
AWARD 3 Umpierrez et al. 2014^20^	DU 0.75 mg (242)	26	7.6	−0.71^	93	−1.36
	DU 1.50 mg (233)		7.6	−0.78^	92	−2.29
	GLA (226)		7.6	−0.56	92	−2.22
	MET dose ≥1,500 mg					
AWARD 4 Jendle et al. 2014^21^	DU 0.75 mg (293)	26	8.5	−1.59^	91.1	0.18**
	DU 1.50 mg (295)		(average for all groups)	−1.64^	(average for all groups)	−0.87**
	GLA (296)			−1.41		2.33
	MET dose ≥1,500 mg					
	GLA dose titrated per protocol					
	(mean 65 U)					
	Lispro dose at endpoint:					
	97 U for DU 0.75 mg					
	93 U for DU 1.5 mg					
	68 U for GLA					
AWARD 5 Nauck et al. 2014^22,23^	DU 0.75+MET (268)	52	8.2	−0.87^	86	−2.60^*
	DU 1.5+MET (258)		8.1	−1.10^	87	−3.03^*
	SITA 100+MET (270)		8.1	−0.39	86	−1.53
	PBO+MET (124)		8.1		87	
	MET dose ≥1,500 mg					
AWARD 6 Dungan et al. 2014^24^	DU 1.5 mg+MET (269)	26	8.1	−1.42^	93.8	−2.90
	Lira 1.8 mg+MET (269)		8.1	−1.36	94.4	−3.61^
	MET dose ≥2,000 mg					

DU = dulaglutide; GLA = Insulin glargine; Lira = liraglutide; MET = metformin; PBO = placebo; PIO = pioglitazone; SIT = sitagliptin; *DU significant versus PBO; ^DU superior versus active comparator; **DU significant versus GLA; ^§^DU noninferior to active comparator.

The advantages of albiglutide include once-weekly dosing and fewer gastrointestinal side effects than the GLP-1 RA liraglutide, but it is less effective at reducing HbA_1c_ and body weight than liraglutide is. On the other hand, it is effective, safe and approved for patients who have renal impairment.^16,17^

## Dulaglutide

Dulaglutide has two human GLP-1 analogues fused to an immunoglobulin (Ig)-G fragment via two short peptide linkers. The half-life after injection amounts to ~90 hours. It is a clear solution, injected with a pen device. In clinical trials, HbA_1c_ reductions and weight loss typical for a GLP-1 RA were observed. Dulaglutide was tested in clinical phase II and III studies in the AWARD study programme. These trials had a similar design, testing two doses of dulaglutide (0.75 mg and 1.5 mg once weekly versus placebo or active comparator for 26–104 weeks). In total, the study population included in this programme comprised more than 5,000 patients, among them more than 3,000 patients on dulaglutide in monotherapy, dual therapy and complex treatment regimes, including insulin.^17–24^ The results from the AWARD studies 1–6 are published.^17–24^ The mean baseline HbA_1c_ for the study population ranged from 7.6 % to 8.5 % and the mean baseline body weight from 86 kg to 96 kg.^17–24^ An overview of the comparative clinical study programme with dulaglutide is given in *Table 2*.

The AWARD studies 7–9 are still ongoing, with results expected soon (AWARD 7: a study in patients with renal insufficiency dulaglutide vs. insulin glargine with insulin lispro in both study arms [ClinicalTrials.gov identifier: NCT01621178], AWARD 8: dulaglutide vs. placebo as add on to sulfonylurea [ClinicalTrials.gov identifier: NCT01769378], AWARD 9: dulaglutide vs. placebo as add on to metformin and insulin glargine [ClinicalTrials.gov identifier: NCT02152371]). In a direct comparison, the add -on of dulaglutide to patients failing on metformin was non-inferior compared with the addition of insulin glargine at the 0.75 mg dose and superior at the 1.5 mg dose.^4^ A direct head-to-head study comparing liraglutide with dulaglutide (in their respective maximal doses approved for type 2 diabetes therapy) showed non-inferiority for dulaglutide regarding the reduction of HbA_1c_ and body weight.^17,24^ The incidence of hypoglycaemic episodes and the adverse event profile was similar to other GLP-1 RAs.

**Figure 1:**
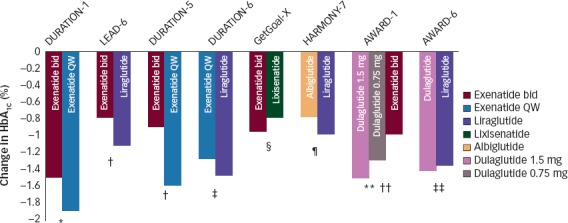
HbA_1c_ Reductions with GLP-1 RA in Head-to-head Clinical Studies p Values are for statistical superiority unless otherwise noted as non-inferiority; *p<0.0025; †p<0.0001; ‡p=0.02; ^§^p=not significant, non-inferiority p value not reported (95 % confidence interval 0.033–0.297, meeting pre-defined non-inferiority margin); ^¶^non-inferiority p value = 0.846 (not meeting pre-defined non-inferiority margin); **p<0.001 for both doses of dulaglutide versus exenatide bid; ††p=not significant, non-inferiority p value <0.0001 (meeting pre-defined non-inferiority margin). HbA_1c_ = glycated haemoglobin. Reproduced with permission from Trujillo et al., 2015.^17^

**Figure 2:**
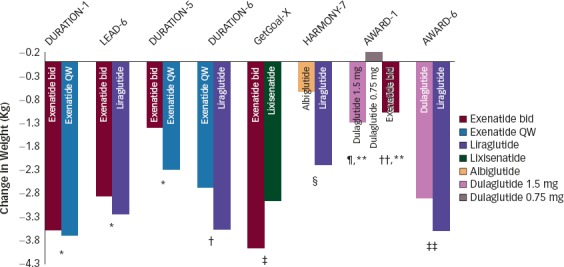
Body Weight Reductions with GLP-1 RA in Head-to-head Clinical Studies p Values are for statistical superiority (unless noted for non-inferiority); *p=not significant; †p=0. 0005; ‡p value not reported for weight difference of 1.02 kg (95 % confidence interval 0.456–1.581); ^§^p<0.0001; ^¶^p<0.001 versus dulaglutide 0.75 mg; **p=not significant between dulaglutide 1.5 mg versus exenatide bid; ‡‡p=0.011. GLP-1 RA = glucagon-like peptide-1 receptor agonists. Reproduced with permission from Trujillo et al., 2015.^17^

## General Considerations about Long-acting GLP-1 Receptor Agonists

The two novel long-acting GLP-1 RAs offer patients who have type 2 diabetes the advantage to lower the glycaemic parameters HbA_1c_ and fasting and postprandial blood glucose while having a low risk of hypoglycaemia as well as the possibility of losing body weight. Generally the gastrointestinal side effects (fullness and nausea) associated with GLP-1 RAs are less severe and less sustained than those of the short-acting GLP-1 RAs. Head-to-head studies between albiglutide and dulaglutide have not been performed so far. The percentage of patients reaching their glycaemic targets during therapy with GLP-1 RAs has been greater than that achieved with most established therapies for type 2 diabetes mellitus. *Figures 1* and *2* show the changes in head-to-head studies with GLP-1 RA for HbA_1c_ (see *Figure 1*) and body weight (see *Figure 2*).^17^

The long-acting GLP-1 RAs activate the GLP-1 receptor continuously, in contrast to the short-acting ones. The pharmacokinetic differences between these drugs lead to important differences in their pharmacodynamic profiles. The short-acting GLP-1 RAs mainly lower the post-prandial plasma glucose through inhibition of gastric emptying, whereas the long-acting compounds have a stronger effect on fasting glucose concentrations, which is mediated mainly through their insulinotropic and glucagonostatic actions. The adverse effect profiles of these compounds also differ. The individual properties of the various GLP-1 RAs might allow incretin-based treatment of type 2 diabetes mellitus to be tailored to each patient’s needs.^25^ The once-weekly dosing interval of the novel GLP-1 RA may be an advantage for patients who present barriers to a once-daily injectable therapy and may give patients also more freedom to live a flexible life without daily therapeutic actions.

GLP-1 RA therapy may have beneficial effects beyond the glycaemic effects by direct action on the endocrine pancreas thanks to the widespread expression of GLP-1 receptors. These may include cardiovascular effects, effects on lipid metabolism, neurological disorders and beneficial effects on systolic blood pressure and body weight. These beneficial effects need to be counterweighed against possible side effects (e.g. gastrointestinal side effects, the increase of pulse rate observed with GLP-1 RA therapy). Data from the ongoing long-term safety studies are needed to judge whether the beneficial effects seen in preclinical and clinical trials will also improve long-term outcomes in the long run. Studies are also ongoing to elucidate the effects of GLP-1 RA in the treatment of type 1 diabetes in conjunction with insulin therapy, in which instance the effects of the GLP-1 RA on gastric emptying and glucagon secretion may have beneficial effects on glycaemic control.^25,26^

## Liraglutide for the Treatment of Obesity

A novel indication for the GLP-1 RA liraglutide is the treatment of obesity. Previous pivotal studies have already demonstrated a significant and sustained body weight reduction with liraglutide injected once daily at doses greater than the 1.2 mg and 1.8 mg used for diabetes.^27^ A large set of prospective clinical studies with more than 3,000 participants has subsequently investigated the effect of 3.0 mg liraglutide daily on the reduction of body weight, cardiovascular effects and safety. In these studies, liraglutide demonstrated a significantly better body weight reduction with 3.0 mg than with a 1.8 mg dose and with placebo. Indeed, 65 % of patients treated with the high dose of liraglutide lost more than 5 % of their body weight. In participants who had type 2 diabetes, the glycaemic effects of both doses were comparable, whereas in study participants who did not have diabetes but who had prediabetes and obesity, the diabetes progression was retarded with use of the high dose of liraglutide. The reduction of blood pressure and the increase in pulse rate were comparable for both liraglutide doses. An improvement of cardiovascular surrogate risk markers was observed for C-reactive protein (CRP), for the lipid parameters and brain natriuretic peptide (BNP). Non-biochemical risk parameters, symptoms and scores for sleep apnoea also improved. All effects of liraglutide were reversible, as observed after a 12-week washout phase at the end of the study.^28–30^

The most common side effect of the 3.0 mg dose of liraglutide was nausea (39 % versus 14 % in the 1.8 mg dose group) and other gastrointestinal side effects characteristic of the GLP-1 RA. Hypoglycaemia was more frequent in the patients who had type 2 diabetes and concomitant sulphonylurea therapy, despite the reduction of the sulphonylurea dose by 50 % (15 % versus 6 %). During the study, seven cases of pancreatitis were observed in the liraglutide group and one case was detected in the placebo group. The incidence of gallstone complications was also higher in the liraglutide group (2.3 % versus 0.9 %).^28–30^

The US Food and Drug Administration (FDA) has approved the 3.0 mg dose of liraglutide for the treatment of obesity. The specific indication for the use of liraglutide in obesity is as an adjunct to lifestyle for chronic weight management in individuals who have a body mass index (BMI) of >30 kg/ m^2^ (corresponding to the diagnostic criteria of obesity) or for subjects who are overweight and who have a BMI >27 kg/m^2^ or greater in the presence of at least one weight-related comorbidity, such as hypertension, diabetes or dyslipidaemia. Patients should be evaluated after 16 weeks and the drug discontinued if the patient has not lost at least 4 % of baseline body weight.

With the approval for the indication obesity for liraglutide, uses for GLP-1 RAs have widened beyond diabetes. The results from the cardiovascular safety and outcome studies lately performed with GLP-1 RA will also answer important questions about the effects of GLP-1 RA and body weight-lowering drugs on cardiovascular risk and on safety. The study results so far show that a higher dose of a GLP-1 RA seems to be safe in terms of fears about increased pulse rate and increased risk of pancreatitis. That progression of diabetes is decreased in subjects with prediabetes on treatment with liraglutide is an interesting finding deserving of more attention in further studies of type 2 diabetes prevention.
